# The Health of the Nation—A Review
1*The Health of the Nation*. By F. E. Fremantle, M.P., M.B., M.Ch., F.R.C.P., F.R.C.S., D.P.H. Pp. xiv., 209. London: Philip Allan & Co. 1927. Price 8s. 6d. net.


**Published:** 1927

**Authors:** 


### THE HEALTH OF THE NATION[1]—A REVIEW.

By The Editor.

---

It is greatly to the reader's advantage that Colonel Fremantle is a practised writer as well as a Member of Parliament and a medical officer of health. Those of us who have enjoyed his former book, *A Doctor in Khaki*, will approach his latest volume with a bias in its favour. But it requires no preconceived good opinion of the author to make his *Health of the Nation* interesting. At the present time, when the health services and the hospitals of this country are the objects of much public discussion, it is obvious that the majority of people who express vigorous and partisan views would cut a poor figure if called upon to pass an examination in the subjects with which Colonel Fremantle's book deals. This small and readable volume presents a complete survey of the way in which the people's health in England, Scotland and Wales is protected. The Minister of Health, Mr. Neville Chamberlain, says in the Foreword : "I welcome this book as a useful and comprehensive review of the organisation and purpose of our public health service." The term public health service is not intended to bear the narrow meaning of the portion of medical work that is entrusted to medical officers of health, for this book discusses almost every provision made by private or public endeavour for the health of the individual and the community. The author is brief, clear and scarcely ever partisan, although in his chapter on industrial conditions he claims a long series of enactments for the credit of the Tory or Conservative Party. But, as the impartial observer may have noticed that the English people hardly permits any legislation to be carried into effect except when a Conservative Government is in power, the apportionment of credit is not germane to the discussion. From time to time public opinion has demanded legislation in the interests of the public health. Sometimes the demands of the public have been readily met by private charity, as in the case of our voluntary hospitals, at other times

[1] *The Health of the Nation.* By F. E. Fremantle, M.P., M.B., M.Ch., F.R.C.P., F.R.C.S., D.P.H. Pp. xiv., 209. London: Philip Allan & Co. 1927. Price 8s. 6d. net.

it has been determined by Parliament that the work demanded by public opinion falls properly into the province of the Guardians of the Poor, or of City or County Health Committees. One important department, that of medical research, has even been placed under the direct control of the Privy Council. All these matters are put out in epitomised form by the author. He has no axe to grind, nor is he advocating some sweeping alteration of our whole system, although he remarks, as any well-informed person must, that our present system is no system at all, but a chance collection of disconnected efforts for the common wealth and health. It needs no great measure of prophetic wisdom to forecast that some co-ordination of these separate and occasionally conflicting efforts is bound to come. We have seen our Navy evolved out of mere gatherings of merchant shipping armed in haste for particular occasions, our Army from bands of noblemen's private retainers, and our Post Office from the attempts of private carriers to serve the public needs. It should cause no astonishment if private enterprise in the care of the nation's health is supplemented or replaced by public undertakings. Colonel Fremantle does not envisage a time when the voluntary system of hospital management will be abandoned, he agrees with the findings of Lord Cave's Committee in 1920, that the voluntary system "with its greater efficiency and economy, its gratuitous services, especially in the training of doctors and nurses, and the sympathy it evokes from the public" is well worth saving. He does not imagine that the responsibilities of other government departments, such as that of the Board of Trade for health in ships and in mines, the Admiralty and the War Office for health in the fighting services, and the Board of Education for health in schools can be transferred with advantage to any one minister. "But," he writes, "a proper revision of the health functions of all government departments and the institution of an effective liaison between them in a common effort under a common strategy, directed by the Minister of Health, is an imperative necessity of the times." This conclusion surely has the assent of everyone who is concerned for the health of the nation. We feel constrained to add that in our opinion the task of carrying such a liaison into effect could not be entrusted to any Minister who commands the confidence of the medical profession more completely than the present one, the Right Hon. Mr. Neville Chamberlain. If changes are to come we hope it may fall to his lot to direct and control them.

